# Supplementary material for: BATSY4-PRO: an open-source multichannel ultrasound recorder for field bioacoustics
Source: BMC Ecol Evol. 2026 Sep 26;26:85. doi: 10.1186/s12862-026-02592-y (PMC13644156; doi:10.1186/s12862-026-02592-y)
Supplement: Supplementary file 1 [file 12862_2026_2592_MOESM1_ESM.pdf]

SUPPLEMENTARY INFORMATION

BATSY4-PRO: An Open-Source Multichannel Ultrasound Recorder for Field Bioacoustics

Ravi Umadi [1]

[1] Lehrstuhl für Zoologie, TUM School of Life Sciences, Technische Universität München, Liesel-Beckmann-Str. 4, 85354, Freising-Weihenstephan, Germany

Correspondence: ravi.umadi@tum.de

CONTENTS

A Component Connections . . . 2
B I²S Clock Setting . . . 2
C Ring Buffer in PSRAM . . . 3
D Real-Time Monitoring Implementation Code . . . 5
D.1 Global Variables and Constants . . . 5
D.2 Real-Time Audio Processing . . . 6
D.3 Monitoring Selection . . . 7
D.4 Mathematical Principle . . . 8
D.5 Performance Characteristics . . . 8
E Bill of Material . . . 9
F Field Data - Call Duration Stats . . . 10
G Geometry-to-Hub Conversion . . . 11

LIST OF FIGURES

S1 Distribution of echolocation call durations in the field dataset. . . . 10
S2 Conversion of WAH-*i* Cartesian coordinates into reconfigurable hub settings. . . . 11

LIST OF TABLES

T1 Inter-component Pin Connections . . . 2
T2 Bill of Materials for the Batsy4 Pro Recording System . . . 9
T3 Bill of Materials for the Field Array . . . 9

A COMPONENT CONNECTIONS

Table T1 records the signal-level connections used in the reported build. Module manufacturers sometimes use different labels for the same signal; connections should therefore be made by signal function rather than by the physical ordering of pins on a particular breakout board. All modules share a common ground, and supply voltage must follow the specification of the particular module used.

Table T1: Pin-to-pin connections for each component in the reported Batsy4-Pro build. The two WM8782 boards use separate Teensy I^{2}S input interfaces. The PCM5102A shares the I^{2}S1 clocks and receives the processed monitoring signal on the Teensy transmit-data line.

Component	Component pin or signal	Connected pin or signal	Function and notes
WM8782 ADC 1	MCLK / BCLK / LRCLK / DOUT	Teensy 23 / 21 / 20 / 8	I^{2}S1 clock, word clock, and stereo input data for M1–M2.
WM8782 ADC 2	MCLK / BCLK / LRCLK / DOUT	Teensy 33 / 4 / 3 / 5	I^{2}S2 clock, word clock, and stereo input data for M3–M4.
PCM5102A DAC	BCK / LCK (WS) / DIN	Teensy 21 / 20 / 7	I^{2}S1 monitoring output; the tested module derives its clocking from BCK and LCK.
SSD1306 OLED	SDA / SCL	Teensy 18 / 19	I^{2}C display at address `0x3C`.
HOLD button	Signal / common	Teensy 40 / GND	Active-low input using `INPUT_PULLUP`.
TAP button	Signal / common	Teensy 41 / GND	Active-low input using `INPUT_PULLUP`.
Rotary encoder	A / B / switch / common	Teensy 36 / 37 / 38 / GND	Polled quadrature input; the switch cycles among carrier, monitored-input, and output-level controls. Carrier selects 10–85 kHz heterodyne or passthrough; input selects Channels 1–4 or their combined mix.
Microphone M1	Analogue output / supply / ground	ADC 1 left input / supply rail / GND	Channel 1; default monitor input at power-up.
Microphone M2	Analogue output / supply / ground	ADC 1 right input / supply rail / GND	Channel 2.
Microphone M3	Analogue output / supply / ground	ADC 2 left input / supply rail / GND	Channel 3.
Microphone M4	Analogue output / supply / ground	ADC 2 right input / supply rail / GND	Channel 4.
microSD card	Built-in socket	`BUILTIN_SDCARD`	Teensy 4.1 onboard microSD interface; no external signal wiring.

B I^{2}S CLOCK SETTING

```
void setI2SFreqBoth(int freq) {
    // SAI prescaler (must be 1-8)
    int n1 = 4;

    // PLL divider
    int n2 = 1 + (24000000 * 27) / (freq * 256 * n1);
    double C = ((double)freq * 256 * n1 * n2) / 24000000;
    int c0 = (int)C;
    int c2 = 10000;
    int c1 = (int)(C * c2) - (c0 * c2);

    set_audioClock(c0, c1, c2, true);
```

```
    // Set SAI1 (I2S1) and SAI2 (I2S2) clock dividers
    CCM_CS1CDR = (CCM_CS1CDR &
    ~(CCM_CS1CDR_SAI1_CLK_PRED_MASK |
    CCM_CS1CDR_SAI1_CLK_PODF_MASK)) |
    CCM_CS1CDR_SAI1_CLK_PRED(n1 - 1) |
    CCM_CS1CDR_SAI1_CLK_PODF(n2 - 1);

    CCM_CS2CDR = (CCM_CS2CDR &
    ~(CCM_CS2CDR_SAI2_CLK_PRED_MASK |
    CCM_CS2CDR_SAI2_CLK_PODF_MASK)) |
    CCM_CS2CDR_SAI2_CLK_PRED(n1 - 1) |
    CCM_CS2CDR_SAI2_CLK_PODF(n2 - 1);
}
```

Listing 1: [2]S clock configuration for 192 kHz sampling on Teensy 4.1.

C RING BUFFER IN PSRAM

```
const int sampleRate = 192000;
const int bytesPerSample = 2;
const int numChannels = 4;
const int ringBufferSeconds = 5;
const uint32_t samplesPerChannel = sampleRate * ringBufferSeconds;
const uint32_t ringBufferSamples = samplesPerChannel * numChannels;

EXTMEM int16_t ringBuffer[ringBufferSamples];
volatile uint32_t ringWriteIndex = 0;

    // Monitoring defaults
    float carrierFreq = 45000.0f;
    const float twoPi = 6.28318530718f;
    float phase = 0.0f;
    float phaseIncrement = twoPi * carrierFreq / sampleRate;
    uint8_t heterodyneChannel = 1;  // Default monitoring input
    const uint8_t MONITOR_MIX_ALL = 5;
    bool passthroughMode = false;
    float outGain = 0.50f;

    // Fill the ring buffer and generate the real-time monitor output
    void readAndProcessAudio() {
    if (queueL1.available() && queueR1.available() && queueL2.available() && queueR2.available()) {
        int16_t *ch1 = (int16_t *)queueL1.readBuffer();
        int16_t *ch2 = (int16_t *)queueR1.readBuffer();
        int16_t *ch3 = (int16_t *)queueL2.readBuffer();
        int16_t *ch4 = (int16_t *)queueR2.readBuffer();
        static int16_t tempOut[128];

        for (int i = 0; i < 128; i++) {
            ringBuffer[ringWriteIndex++] = ch1[i];
            ringBuffer[ringWriteIndex++] = ch2[i];
```

```
                ringBuffer[ringWriteIndex++] = ch3[i];
                ringBuffer[ringWriteIndex++] = ch4[i];
                if (ringWriteIndex >= ringBufferSamples) ringWriteIndex = 0;

                float monitorInput;
                switch (heterodyneChannel) {
                    case 2: monitorInput = ch2[i]; break;
                    case 3: monitorInput = ch3[i]; break;
                    case 4: monitorInput = ch4[i]; break;
                    case MONITOR_MIX_ALL:
                        monitorInput = (int32_t)ch1[i] + ch2[i] + ch3[i] + ch4[i];
                        break;
                    default: monitorInput = ch1[i]; break;
                }

                float monitorSample = monitorInput * outGain;
                if (!passthroughMode) {
                    monitorSample *= sinf(phase);
                    phase += phaseIncrement;
                    if (phase >= twoPi) phase -= twoPi;
                }
                if (monitorSample > 32767.f) monitorSample = 32767.f;
                if (monitorSample < -32768.f) monitorSample = -32768.f;
                tempOut[i] = (int16_t)monitorSample;
            }
            totalSamplesWritten += 128 * numChannels;

            queueL1.clear();
            queueR1.clear();
            queueL2.clear();
            queueR2.clear();

            if (outputQueue.available()) {
                int16_t *out = outputQueue.getBuffer();
                memcpy(out, tempOut, sizeof(tempOut));
                outputQueue.playBuffer();
            }
        }
    }


    // On trigger, dump ring buffer to SD card
    void dumpRingBufferToSD() {
        startNewRecording();  // open temp file
        uint32_t idx = ringWriteIndex;
        for (uint32_t i = 0; i < ringBufferSamples; i++) {
            int16_t sample = ringBuffer[idx++];
            file.write((uint8_t*)&sample, 2);
            sampleCount++;
            if (idx >= ringBufferSamples) idx = 0;
        }
        closeWavFile();  // insert header and rename file
```

```
    }
```

Listing 2: Ring Buffer Implementation

D REAL-TIME MONITORING IMPLEMENTATION CODE

This appendix provides the key firmware implementation of the selectable real-time monitoring subsystem described in the main manuscript.

D.1 Global Variables and Constants

Heterodyne mode uses a phase accumulator to generate a continuous sine-wave carrier. The carrier selector also provides passthrough states, and the input selector includes the combined four-channel mix:

```
// Heterodyne carrier parameters
const float FREQ_MIN   = 10000.0f;     // Minimum carrier frequency (Hz)
const float FREQ_MAX   = 85000.0f;     // Maximum carrier frequency (Hz)
const float FREQ_STEP  = 5000.0f;      // Frequency adjustment step (Hz)
const int CARRIER_FREQ_COUNT =
    (int)((FREQ_MAX - FREQ_MIN) / FREQ_STEP) + 1;

volatile float carrierFreq = 45000.0f;  // Default carrier: 45 kHz
volatile float outGain     = 0.50f;     // Output gain: 0.0-1.0 (50% default)
uint8_t heterodyneChannel  = 1;         // Default monitored input channel
const uint8_t MONITOR_MIX_ALL = 5;       // Sum of Channels 1--4
const uint8_t MONITOR_SELECTION_COUNT = 5;
bool passthroughMode = false;            // PT bypasses heterodyning
int carrierSelection =
    1 + (int)((45000.0f - FREQ_MIN) / FREQ_STEP); // Default: 45 kHz
const float VOL_STEP   = 0.05f;         // Volume adjustment step (5%)

// Phase accumulator variables
const float twoPi = 6.28318530718f;     // 2*pi constant
float phase = 0;                        // Current phase position [0, 2*pi]
float phaseIncrement = 0;               // Phase increment per sample

// Audio system parameters
const int sampleRate = 192000;          // Sampling rate: 192 kHz
const int numChannels = 4;              // Number of input channels
```

Listing 3: Global variables for selectable real-time monitoring.

The phase increment is calculated during initialisation based on the carrier frequency and sampling rate:

```
void setup() {
    // ... other initialisation code ...

    // Calculate phase increment for carrier generation
    phaseIncrement = twoPi * carrierFreq / sampleRate;

    // For 45 kHz carrier at 192 kHz sampling:
    // phaseIncrement = 6.283 * 45000 / 192000 = 1.472 radians/sample

    // ... rest of setup code ...
}
```

Listing 4: Phase increment initialization in `setup()`.

D.2 Real-Time Audio Processing

The `readAndProcessAudio()` function fills the PSRAM ring buffer with four raw channels, forms the selected monitor input, and sends the monitoring signal to the DAC. Channel 1 is selected at power-up; the operator can select Channels 1–4 or `MX`, which sums all four inputs. Heterodyne mode multiplies this signal by the carrier, whereas `PT` sends it directly to the output stage.

```
void readAndProcessAudio() {
    // Check if all four input queues have data available
    if (queueL1.available() && queueR1.available() &&
    queueL2.available() && queueR2.available()) {

        // Get pointers to 128-sample blocks from each channel
        int16_t *ch1 = (int16_t *)queueL1.readBuffer();
        int16_t *ch2 = (int16_t *)queueR1.readBuffer();
        int16_t *ch3 = (int16_t *)queueL2.readBuffer();
        int16_t *ch4 = (int16_t *)queueR2.readBuffer();

        // Temporary buffer for monitor output
        static int16_t tempOut[128];

        // Process 128 samples (one audio block)
        for (int i = 0; i < 128; i++) {
            // 1. Write raw samples to ring buffer (all 4 channels, interleaved)
            ringBuffer[ringWriteIndex++] = ch1[i];
            ringBuffer[ringWriteIndex++] = ch2[i];
            ringBuffer[ringWriteIndex++] = ch3[i];
            ringBuffer[ringWriteIndex++] = ch4[i];
            if (ringWriteIndex >= ringBufferSamples) ringWriteIndex = 0;

            // 2. Select one channel or sum all four in MX mode
            float monitorInput;
            switch (heterodyneChannel) {
                case 2: monitorInput = ch2[i]; break;
                case 3: monitorInput = ch3[i]; break;
                case 4: monitorInput = ch4[i]; break;
                case MONITOR_MIX_ALL:
                    monitorInput = (int32_t)ch1[i] + ch2[i] + ch3[i] + ch4[i];
                    break;
                default: monitorInput = ch1[i]; break;
            }

            // 3. PT passes the input directly; otherwise heterodyne it
            float monitorSample = monitorInput * outGain;
            if (!passthroughMode) {
                monitorSample *= sinf(phase);
                phase += phaseIncrement;
                if (phase >= twoPi) phase -= twoPi;
            }

            // 4. Limit to the 16-bit output range
            if (monitorSample > 32767.f) monitorSample = 32767.f;
            if (monitorSample < -32768.f) monitorSample = -32768.f;
            tempOut[i] = (int16_t)monitorSample;
        }

        // Update total sample counter (for ring buffer readiness check)
        totalSamplesWritten += 128UL * numChannels;

        // Release input buffers
```

```
            queueL1.clear();
            queueR1.clear();
            queueL2.clear();
            queueR2.clear();

            // 5. Send monitor audio to the DAC output (PCM5102A)
            if (outputQueue.available()) {
                int16_t *out = outputQueue.getBuffer();
                memcpy(out, tempOut, sizeof(tempOut));  // Copy 128 samples
                outputQueue.playBuffer();                // Stream to I2S output
            }
        }
    }
```

Listing 5: Selectable real-time monitoring with parallel four-channel recording.

D.3 Monitoring Selection

The rotary-encoder switch cycles among carrier, monitored-input, and output-level controls. The OLED highlights the active control. Carrier selection traverses `PT`, 10–85 kHz, and `PT`; input selection cycles through Channels 1–4 and `MX`:

```
    void applyEncoderSteps(int detents) {
        if (detents == 0) return;

        if (editMode == EDIT_FREQ) {
            carrierSelection += detents;
            if (carrierSelection < 0) carrierSelection = 0;
            if (carrierSelection > CARRIER_FREQ_COUNT + 1)
                carrierSelection = CARRIER_FREQ_COUNT + 1;
            passthroughMode = carrierSelection == 0 ||
                              carrierSelection == CARRIER_FREQ_COUNT + 1;
            if (!passthroughMode) {
                carrierFreq = FREQ_MIN + (carrierSelection - 1) * FREQ_STEP;
                phaseIncrement = twoPi * carrierFreq / sampleRate;
            }

        } else if (editMode == EDIT_VOL) {
            // Adjust output gain (volume)
            outGain += detents * VOL_STEP;  // +/- 5% per detent

            // Clamp to valid range [0.0, 1.0]
            if (outGain < VOL_MIN) outGain = VOL_MIN;
            if (outGain > VOL_MAX) outGain = VOL_MAX;

            Serial.print("Output Volume: ");
            Serial.print(outGain * 100.0f, 0);
            Serial.println("%");
        } else {  // EDIT_HET_CH mode
            // Cycle through Channels 1--4 and MX
            int nextChannel = (int)heterodyneChannel - 1 + detents;
            nextChannel %= MONITOR_SELECTION_COUNT;
            if (nextChannel < 0) nextChannel += MONITOR_SELECTION_COUNT;
            heterodyneChannel = (uint8_t)(nextChannel + 1);
        }

        drawUI();  // Update OLED display
    }
```

Listing 6: Monitoring-mode and input selection via rotary encoder.

These selections change only the signal sent to the monitoring output. The raw samples from all four microphones continue to be written separately to the ring buffer and saved in every recording.

D.4 Mathematical Principle

In heterodyne mode, multiplication in the time domain corresponds to convolution in the frequency domain. Given an input signal $x[n]$ containing a spectral component at frequency f_{bat}, and a carrier sine wave at frequency f_{carrier}, the multiplication produces:

$$y[n] = x[n] \cdot \sin(2\pi f_{\text{carrier}} n / f_s) \cdot g \tag{1}$$

where $f_s = 192\,\text{kHz}$ is the sampling rate and g is the output gain. This operation generates sum and difference frequencies:

$$f_{\text{sum}} = f_{\text{bat}} + f_{\text{carrier}} \tag{2}$$

$$f_{\text{diff}} = |f_{\text{bat}} - f_{\text{carrier}}| \tag{3}$$

The sum frequency is typically above the Nyquist limit (96 kHz) and is naturally filtered by the DAC reconstruction filter. The difference frequency falls into the audible range, enabling real-time monitoring through standard headphones.

Example: A bat echolocation call at 62 kHz mixed with a 60 kHz carrier produces:

- Sum: $62 + 60 = 122\,\text{kHz}$ (inaudible, filtered)
- Difference: $|62 - 60| = 2\,\text{kHz}$ (audible tone)

This 2 kHz tone is heard in real time through the headphones, allowing the researcher to detect bat activity without viewing a spectrogram.

D.5 Performance Characteristics

- **Computational cost:** Per 128-sample block (0.67 ms at 192 kHz):
  - 128 calls to `sinf()` (hardware-accelerated)
  - 128 floating-point multiplications
  - 128 floating-point to integer conversions
  - Negligible impact on Teensy 4.1 @ 600 MHz
- **Latency:** <1 ms (one audio block buffering delay)
- **Frequency response:** Flat across tunable range (10–85 kHz)
- **Dynamic range:** Limited by 16-bit integer output (~96 dB theoretical)
- **Harmonic distortion:** Minimal (depends on `sinf()` precision)
- **Interference:** None—operates in parallel with ring buffer recording

E BILL OF MATERIAL

Table T2: Bill of Materials for the Batsy4 Pro Recording System. The listed prices reflect procurement costs in Germany during development, from January to July 2025.

Item	Description	Count	Unit Cost (€)	Source
Teensy 4.1	Microcontroller	1	39.50	reichelt.de
WM8782	I2S ADC	2	14.90	Audio-phonics.fr
Knowles SPU0410LR5H-QB	MEMS Mics	4	0.50	Digikey.de
APS6404L-3SQR PSRAM - 8MB -	External Memory	2	2.50	tinytronics.nl
PC5102A	I2S DAC	1	7.99	Amazon.de
MP1584EN	DC-DC 3A Buck Converter	1	2.00	Amazon.de
SSD1306	OLED Display	1	5.25	AZDelivery.de
Push Buttons	User Input	2	0.50	Amazon.de
Rotary Encoder	User Input	1	1.50	Amazon.de
Circuit Switch	Power Supply ON/OFF	1	0.25	Amazon.de
Perforated PCB	System Base	3	1.50	Amazon.de
Hexnut Screws	PCB Stacking	Assorted	5.00	Amazon.de
Wiring	18/22 gauge coloured wires	Assorted	5.00	Amazon.de
6-Pin Connector	Mic power and signal connection	1	14.99	Amazon.de
CR2032	RTC Battery	1	1.50	Generic
**Total**			**125.28**	

Table T3: Bill of Materials for the Field Array. The material list is only suggestive; equivalent components may be used depending on array design and local availability.

Item	Description	Count	Unit Cost (€)	Source
Aluminium Square Hollow Profiles (1 cm^2)	Arms of the array	4	4.99	Hagebaumarkt
Aluminium Square Solid Profile (1 cm^2)	Holder, mounting rod	1	7.99	Hagebaumarkt
Ball Joint	Tripod mount (or use 1/4" mount)	1	13.50	Generic
1/4" Tripod Mount Hot Shoe Adapter	Between holder and ball joint	1	5.99	Amazon (generic)
6-way Plastic Connector	Holds the array arms and rod	1	4.99	Hagebaumarkt
Polyester Fur	Acoustic insulation	1 m^2	8.99	Amazon (generic)
Cables and Miscellaneous	Extension cables, adhesives, etc.	N/A	20.00	Local purchases
**Total**			**81.42**	

F FIELD DATA - CALL DURATION STATS

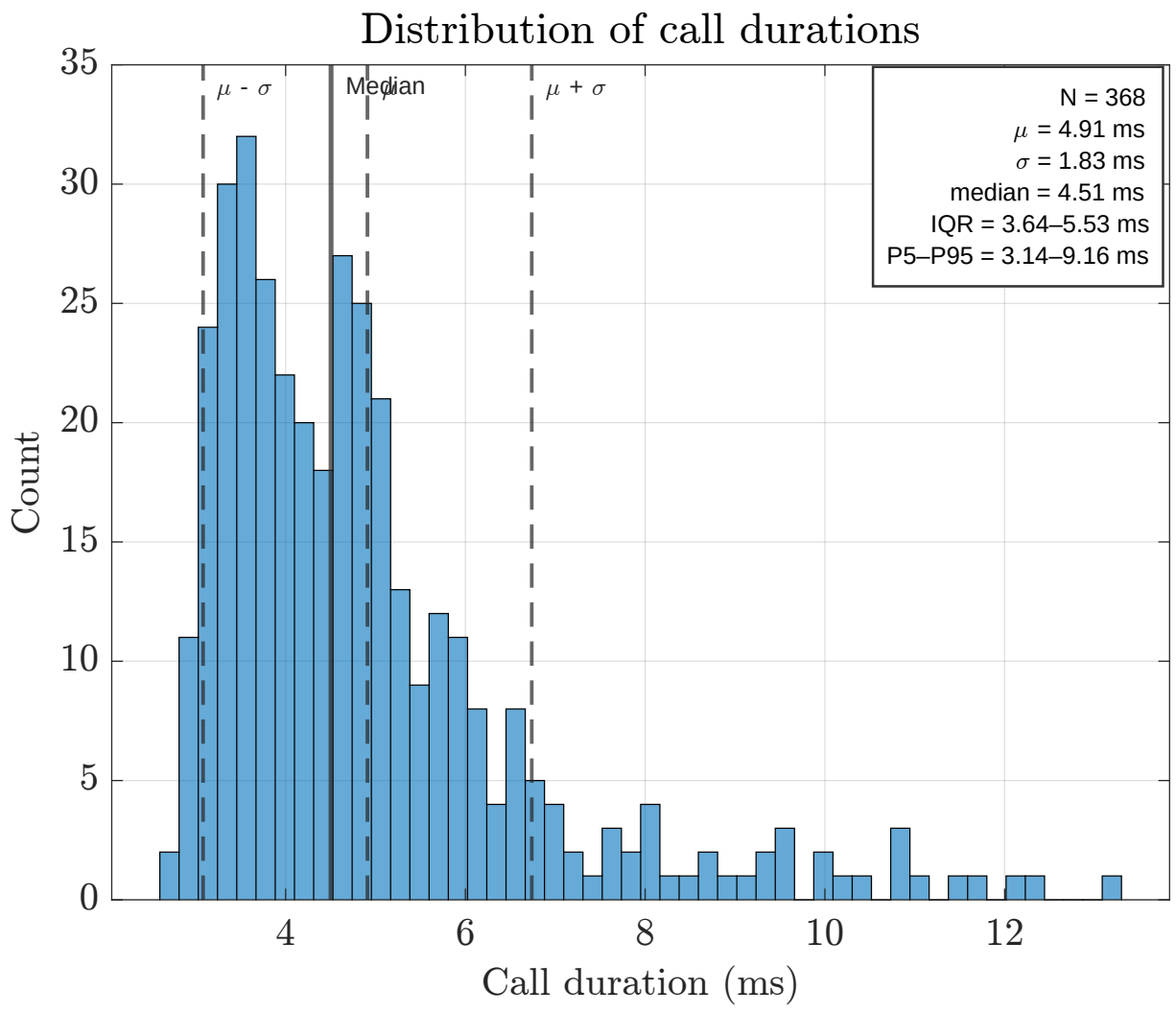

Figure S1: Distribution of echolocation call durations extracted from validated detections ($N = 368$ calls). Call duration was defined using an energy-based percentile criterion: for each call waveform segment, the cumulative sum of squared amplitude was normalised, smoothed, and the call duration was taken as the interval between the time points at which the cumulative energy crossed the lower and upper fractional thresholds (5–95% of total energy). The histogram shows a unimodal distribution centred on short-duration frequency-modulated calls. The vertical solid line indicates the median call duration (4.51 ms), while the shaded region denotes the interquartile range (IQR: 3.64–5.53 ms). Dashed lines mark the 5th and 95th percentiles (3.14–9.16 ms), illustrating the limited spread and absence of long-duration outliers.

G GEOMETRY-TO-HUB CONVERSION

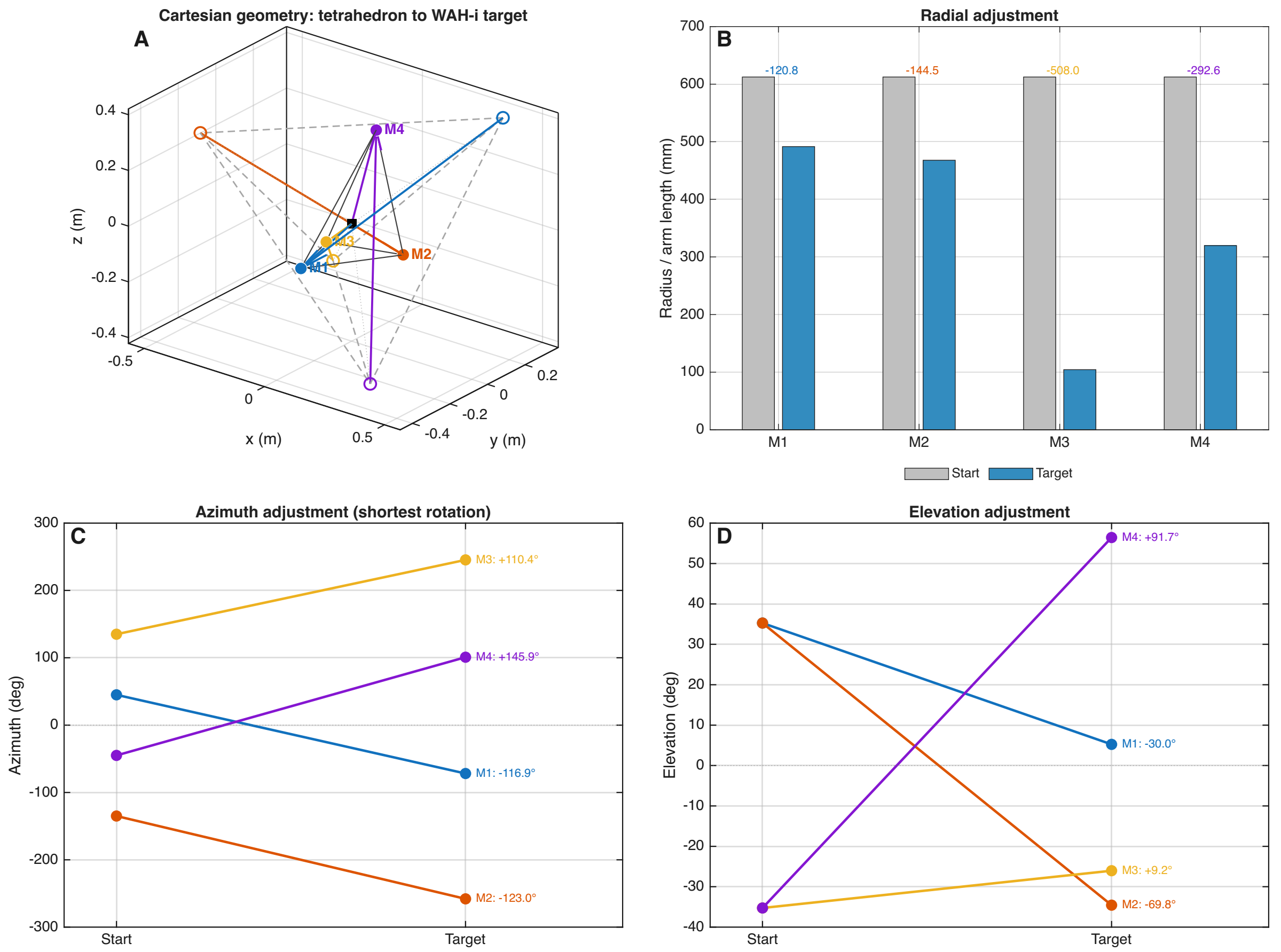

Figure S2: Conversion of an example WAH-*i* Cartesian output into settings for a reconfigurable four-arm hub. (A) Regular tetrahedral starting geometry (open circles and dashed edges; 1 m edge length) and the optimised target geometry (filled circles and solid edges), with arrows indicating microphone displacement. (B) Starting and target radial arm lengths; annotations give the signed length changes in millimetres. (C) Starting and target azimuths, expressed using the shortest signed rotations. (D) Starting and target elevations. Coordinates share a common hub origin, with $+x$ defining zero azimuth, $+y$ positive azimuth, and $+z$ positive elevation; microphone identity is preserved by CSV row order.
